# ‘Self body-management and thinness in youth: survey study on Italian girls’

**DOI:** 10.1186/s12955-018-0937-4

**Published:** 2018-06-08

**Authors:** Dina Di Giacomo, Giulia De Liso, Jessica Ranieri

**Affiliations:** Department of Life, Health and Environmental Sciences, University of L’Aquila, P.le Tommasi, n.1, 67010 L’Aquila, Italy

**Keywords:** Thinness, Girls, Youth, Body satisfaction, Body mass index

## Abstract

Adherence to the thinness model, self-acceptance such as self-esteem is psychological dynamics influencing the young age and emerging adulthood of women life. The purpose of this study was to investigate the girls and young women’ ability to deal with the adherence to thinness model according to their self-body management thought daily self-perception of ownhabits and aptitude. We analysed their emotional patterns and body management to elucidate the Italian phenomenon. A cross-sectional study was conducted on 2287 Italian female distribute in range age 15–25 years old and distributed in girl and young women groups. We conducted a Survey study by snowball sampling technique. Our results showed that girls had higher emotional pattern scores when their weight and shape fit the thinness model: skinny girls felt positively about their body even if when they did not take adequate care of it. Italian girls consider the underweight body mass index an adherence model. Findings suggest the urgent need to plan prevention programme to model healthy behaviours about their daily good practice overcoming social and cultural models based on appearance.

## Introduction

Youth is a crucial growth period in female life for multiple complex domains, including identity formation. This process of growth affects various features, including physical, mental, social, and sexual features. Self-acceptance is a relevant psychological condition for young age and emerging adulthood. Main predictive of wellness in youth is the body satisfaction. Weight and shape are two substantial concerns for young people that dramatically rise throughout their development, so living a continue process of changing. Boys and girl behaviours are influenced by their low self-acceptance, and/or self-esteem, and/or body image acceptance can be predictive factors for negative impact on body satisfaction and self-awareness [1–4].

Body satisfaction is a subjective evaluation, considered the affective component of the multi-dimensional construction of body image [5]. Several researchers have investigated the negative impacts of body dissatisfaction, or displeasure with one’s weight or shape. Girls seem more likely to suffer from body dissatisfaction following puberty; indeed, Runfola et al. [6] suggested that this condition is rather common among females. Moreover, Smink et al. [7] highlighted that a small percentage of children and adolescents with body dissatisfaction engage in disordered eating, which can quickly become a major health concern. High levels of body dissatisfaction and weight concerns are particularly likely to occur during adolescence and early adulthood. Lucena-Santos [8] suggested that psychological flexibility of body image is a mediator of lasting pathological implications (i.e., physical and mental disorders) of body dissatisfaction. According to Hill et al. [9], body image flexibility is a protective factor against eating disorders in girls with low body mass index (BMI). In fact, among girls and women, being underweight is a physical risk factor of such disorders, and is even more detrimental if it develops during an early age.

The strong sociocultural emphasis on appearance has been considered as a major risk factor of adherence to the thinness model, which can propel adolescents and youth into a dysfunctional life and behavioural style, such as taking dramatic measures to alter their appearance, cosmetic surgery, and steroid use. The meta-analysis of Dittmar & Howard [10] investigated the impact of thinness as cultural ideal of female beauty. The researches evidenced the exposition to the thin model appeared being a negative factor for mental health of women: they tend to feel worse their bodies, and then develop body dissatisfaction and psychological weakness. These are all associated with emotional distress and psychiatric disturbances, such as depression and eating disorders [11–13]. Pathological behaviour patterns can be affected by psychological and behavioural problems with more lasting effects [14, 15].

Few studies have sought to understand the factors that increase the risk of body image concerns among girls and women [16]. Clay et al., [17] highlighted the adolescence as age particularly sensible but not vulnerable to the sociocultural factors in the body satisfaction and self-esteem. Puharic et al. [18] have studied in depth the factors influencing the attitudes of adolescences about their body and appearance satisfaction: findings have evidenced as skinny girls are less sensible to the appearance as social standard. So far, the research interests have been focused on the external variables influencing the self-perception and wellness.

Thus, the aim of the present study was to investigate how behavioural attitudes relate to the self-body management of youth and emerging adulthood. Specifically, we wanted to analyse the relationship between emotional pattern and body management, according the age and BMI. Aim was to provide evidence and observe trends in wide range age including young girls and emerging adults; we intended to investigate the self-management of body satisfaction along 2 sensible periods of life of female youth when the body satisfaction and the self-acceptance represent key indexes of quality of life. We detected the Italian girls’ cognitions and behaviours related to adherence to thinness and appearance needs by conducting a Survey study on large-scale evaluation.

## Method

### Participants

Participants were 2287 Italian girls aged 15–25 years old (*M* = 22.2, *SD* = 2.11). All participants were living in the North (*N* = 832; 36.4%), Middle (*N* = 712; 31.1%), or South of Italy (*N* = 743; 32.5%). The participants have been distributed in n. 2 groups assuming the age 20 as threshold: a) Girls group was composed of n. 486 young (*M* = 19,1, *SD* = 1,1); b) Young Women group composed of n. 1801 women (*M* = 23,1, *SD* = 1,4). Among Girls, 188 (8.2%) were living in the North of Italy, 172 (7.5%) in the Middle, and 126 (5.5%) in the South; among Young Women, 644 (28.2%) were from the North, 540 (23.6%) from the Middle, and 617 (27%) from the South. This distribution covers all living area (rural, underground or metropolitan areas).

Weight and height data were detected to calculate BMI (weight in kilograms divided by squared height in meters) and we divided the Girls and Young Women groups into 7 subgroups of BMI based on the criteria of the World Health Organization: severely underweight (BMI < 16.00), underweight (BMI 16.00–18.49), normal (BMI 18.50–24.99), overweight (BMI 25.00–29.99), obese class 1 (BMI 30.00–34.99), obese class 2 (BMI 35.00–39.99), and obese class 3 (BMI ≥40.00). In Table 1 were reported demographic data of the participants.

**Table 1:** Descriptive statistics of ‘self-perception and behavior’

Report				
BMI groups		Emotional pattern score	Body managing score	Self-perception and behavior score
Severely underweight	Mean	6,58	8,50	15,08
	ds	2151	1446	3232
Underweight	Mean	7,01	9,24	16,26
	ds	1951	1239	2529
Overweight	Mean	5,65	9,20	14,84
	ds	1832	1226	2369
Normal	Mean	6,19	9,00	15,19
	ds	1892	1202	2311
Obese Class 1	Mean	5,68	9,20	14,88
	ds	1851	1314	2087
Obese Class 2	Mean	5,18	9,21	14,39
	ds	1960	1536	2999
Obese Class 3	Mean	5,25	9,71	14,94
	ds	2017	,772	2380
Total	Mean	6,16	9,07	15,23
	ds	1926	1221	2381

Recruitment was carried out using social media involving mainly medical doctors. The inclusion criteria were: a) female, b) aged 15–25 years; and c) gave written informed consent.

### Instruments

Trained medical doctors collected the socio-demographic characteristics of participants, such as age, residential area, weight and height, using a socio-demographics inventory. A self-report questionnaire was used to analyse psychological factor of body satisfaction and the related body management.

### Body satisfaction and behaviour

**Fig. 1:**
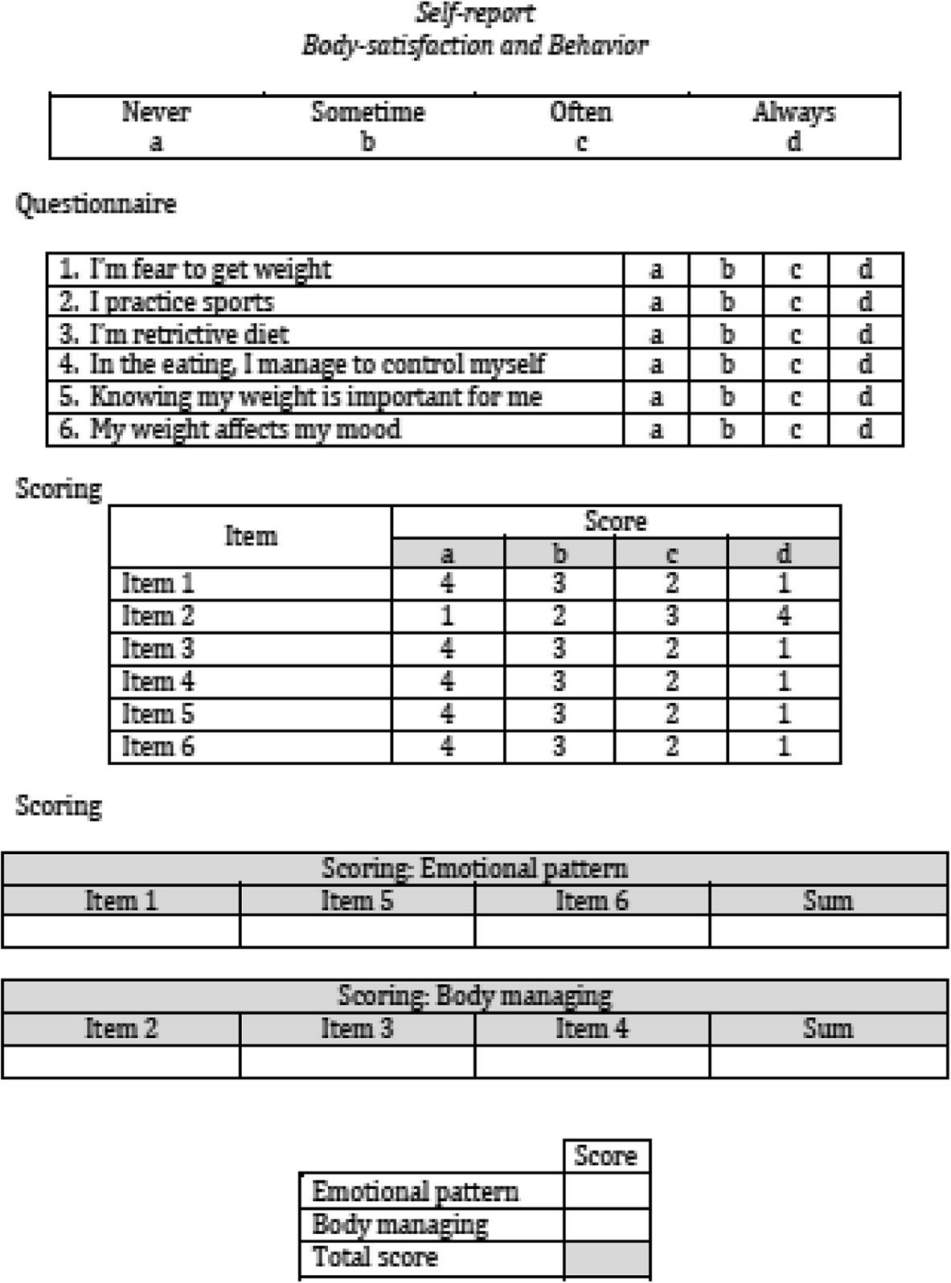
Self report: questionnaire and scoring procedure

We used an experimental self-report questionnaire containing two factors: a) emotional pattern and b) body management. Both variables were rated on a four-point Likert-type scale ranging from ‘never’ to ‘always’. The entire scale consisted of 6 items equally divided between the two factors. Figure 1 shows the self-report questionnaire and scoring procedure.

The ‘emotional pattern’ factor assessed participants’ feelings about their own weight, and was composed of 3 items: the fear of gaining weight (Item 1), importance of knowing their own weight (Item 5), and the influence of weight on mood (Item 6). The ‘body management’ factor assessed participants’ behaviour in taking care of their own body to manage its shape, consisting of 3 items: frequency of sport practice (Item 2), adoption of a restrictive diet (Item 3), and self-control when eating (Item 4). The self-report questionnaire has been developed by pilot study: we arranged a longer questionnaire and experimented it in pilot study previously carried out on sample not included in this research; the final version applied in study has been the result of the elaboration data and editing process: finally the internal reliability the Body Satisfaction and Behaviour report was was good (Cronbach α=0.91).

### Procedure

The individuals were recruited using the snowball sampling technique, a non-randomized method of sample selection. Participants were contacted using social media (Facebook).

In first time, we involved medical doctors in the study my mailing enrolling. They were trained about the collection of BMI index and then they recruited the eligible participants.

Our staff provided a digital and online form of self-report. Afterward, the self-report questionnaire was linked on the Facebook post, and participants could access it after giving written informed consent. Informed consent was obtained from each participant, and the study adhered to the Declaration of Helsinki. Girls less than 18 years old were also asked to deliver the informed consent form to their parents, who had to then decide if they would consent to their adolescent taking part in the study. Young Women 18 years old or older were asked to provide informed consent themselves.

The online self-report questionnaire could be completed in about 15 min; after filling it in, participants submitted it online. The data were collected into dedicated server. The responsible of sensible data was Prof. Di Giacomo (head of project).

### Statistical analyses

All data were carefully double-checked for possible miscoding, the distribution of values, and updating of missing values prior to analysis (some items had missing data, which we replaced using the series mean method).

The participants were grouped according to BMI and age range. Descriptive statistics, multivariate analysis of variance (MANOVA), and the least significant different (LSD) test (as a post hoc test) were carried out using SPSS Statistics 22.0. The significance level was fixed at α < 0.05.

## Results

### Statistical analyses were conducted on collected data

Table 2 shows the distribution of both groups Girls and Young Women by BMI categories. There was a high frequency in the normal category (69.6%) and low frequencies in the underweight (10.4%) and overweight (14.1%) categories.

**Table 2:** Demographic data of participants

Age group	MBI group	Number	Percent
Adolescent	Severely underweight	5	0,2%
	Underweight	74	3,2%
	Normal	320	14,0%
	Overweight	67	2,9%
	Obese Class 1	12	0,5%
	Obese Class 2	6	0,3%
	Obese Class 3	2	0,1%
	Total	486	21,3%
Young	Severely underweight	7	0,3%
	Underweight	164	7,2%
	Normal	1271	55,6%
	Overweight	255	11,1%
	Obese Class 1	62	2,7%
	Obese Class 2	27	1,2%
	Obese Class 3	15	0,7%
	Total	1801	78,7%
Total	Severely underweight	12	0,5%
	Underweight	238	10,4%
	Normal	1591	69,6%
	Overweight	322	14,1%
	Obese Class 1	74	3,2%
	Obese Class 2	33	1,4%
	Obese Class 3	17	0,7%
	Total	2287	100,0%

A MANOVA (3 × 7 × 2) was conducted to examine differences in body satisfaction variables according to BMI and age. Wilks’ lambda indicated a significant effect of BMI group (F(1,6) = 7.45; *p* = 0.001; η^2^ = 1.00), but there was no effect of age group or an interaction. The within-subjects effect tests indicated significant differences in emotional pattern (*p* < .001; η^2^ = 1.00) and body management (*p* < .007; η^2^ = 0.90), as well as in the overall score for body satisfaction and behaviour (*p* < .001; η^2^ = 1.00).

**Fig. 2:**
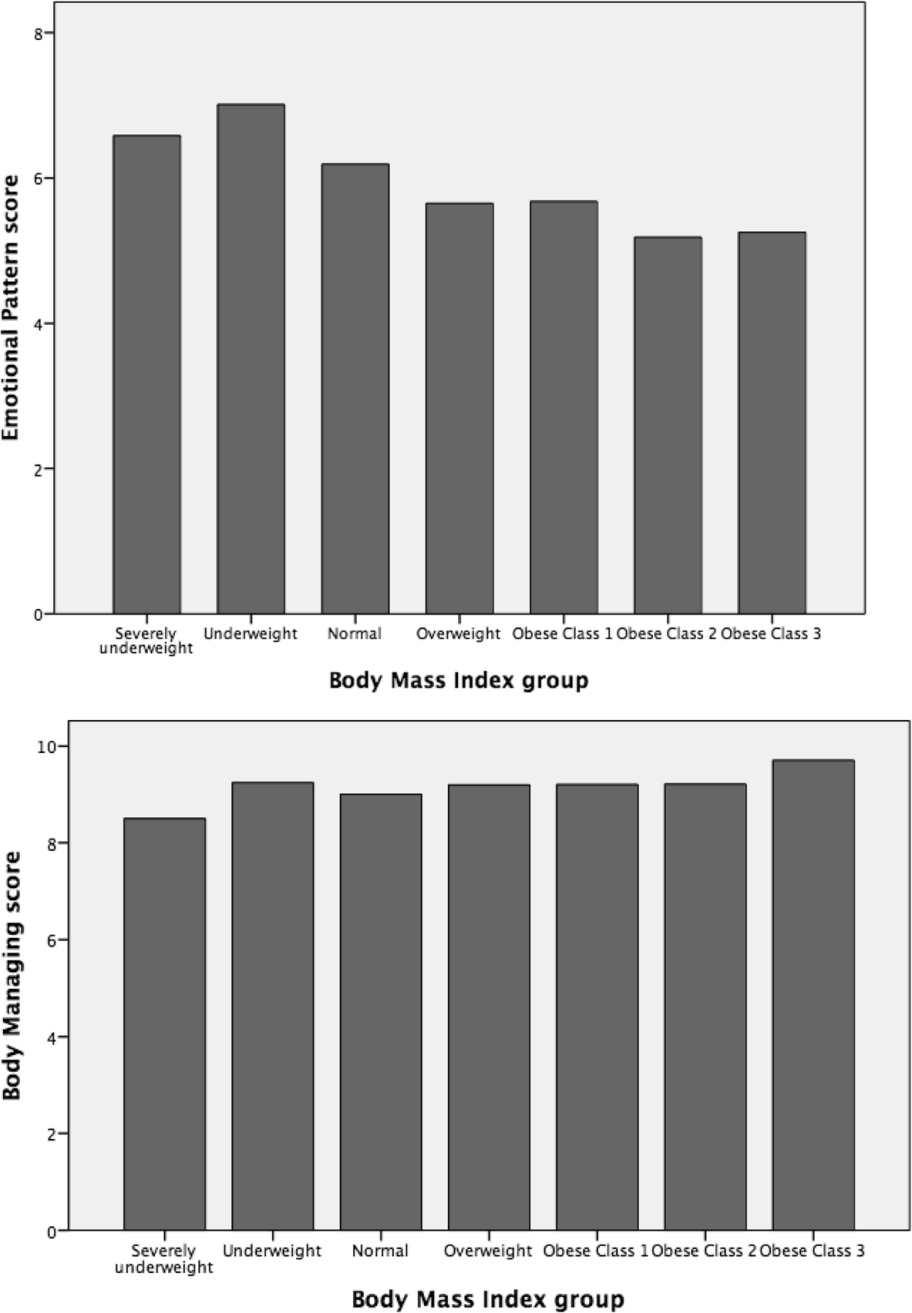
Representation of Emotional pattern and Body managing trends

**Fig. 3:**
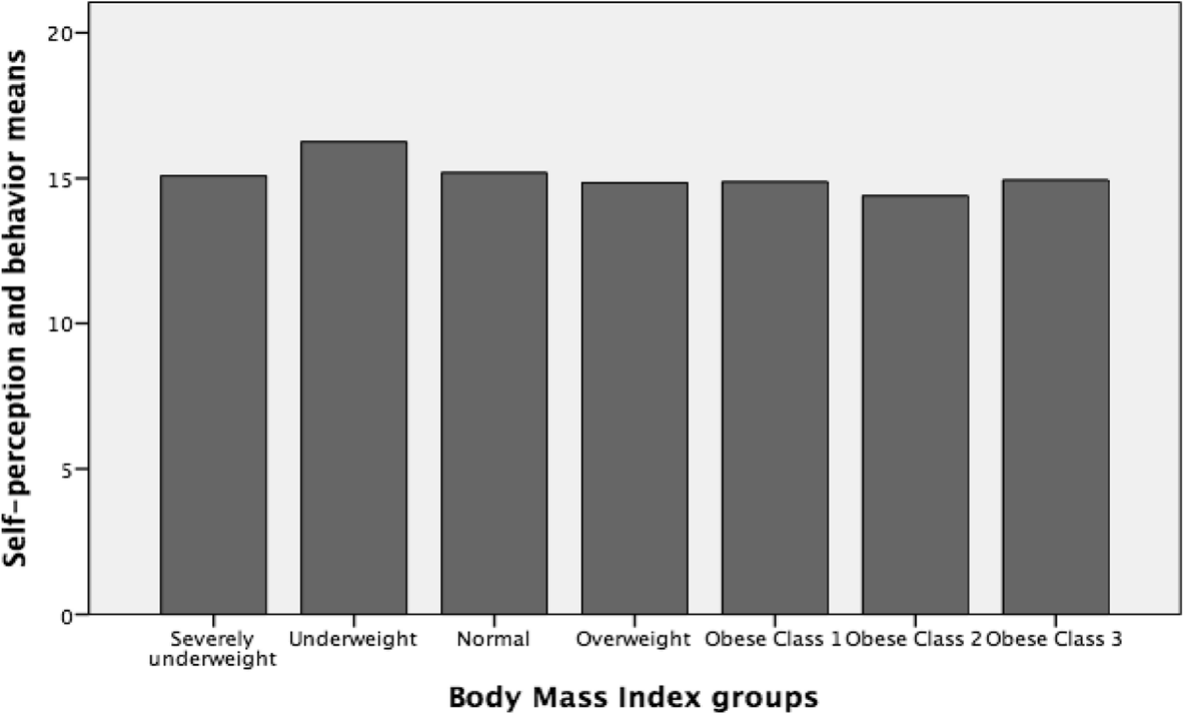
Representation of ‘Self-perception and behavior’ means among BMI groups

Figures 2 and 3 show the distributions of the examined variables.

Post-hoc analyses (using the LSD test) revealed significant differences in the emotional pattern variable for the following comparisons: severely underweight vs. obese class 2 (*p* = 0.02); underweight vs. normal (*p* = 0.001, overweight (*p* = 0.000), obese class 1 (*p* = 0.001), and obese class 3 (*p* = 0.001); normal vs. overweight (*p* = 0.001), obese class 1 (*p* = 0.02), and obese class 2 (*p* = 0.002). As for the body management variable, significant differences were found for normal vs. underweight (df = − 025; *p* = 0.004), overweight (df = − 0.20; *p* = 0.009), and obese class 3 (df = − 0.21; *p* = 0.02), and for severely underweight vs. underweight (*p* = 003).

Finally, the total body satisfaction and behaviour score significantly differed in the comparison of underweight vs. overweight (*p* = 0.000), normal (*p* = 0.001), obese class 1 (*p* = 0.001), obese class 2 (*p* = 0.001), and obese class 3 (*p* = 0.03), as well as for normal vs. overweight (*p* = 0.01).

## Discussion and conclusion

The study aimed to analyse how the female’ body satisfaction and related behaviours differed according to age and BMI, in Italian young and emerging adulthood population.

Our results showed that participants exhibited higher emotional pattern scores (i.e., positive feelings about their own body) when their weight and shape corresponded to thinness model. Particularly, skinny girls felt positively about their body even if they did not manage to take care of their body adequately (i.e., less sports engagement and a more restrictive diet). This is an important point: females, both in youth or emerging adulthood, tend to consider themselves positively when skinny but do not focus much attention on their improving health. According Dittmar & Howard [10], when the weight and shape of body image reflect thin image ideal can have positive psychological effect; nevertheless, in our opinion that process highlighted hazardous living habits because their behaviours don’t be suggested by healthy daily practice; rather, manage their appearance only due to aesthetic or social reasons. Conversely, girls with a normal BMI, despite being in good shape and having a body that fits positively to the thinness model, had negative emotional pattern and engaged in less body management. The normal group also seemed to exhibit more negative body management behaviours when compared with the overweight group. Finally, the overweight and obese groups displayed highly negative emotional patterns but were more focused on managing their bodies. Regarding the overall body satisfaction and behaviour index, scores were higher for the underweight group than others. Surprisingly, no age differences were found, suggesting the need for urgent educational interventions focused on better managing the health of the overall female population in order to reduce the risk of developing future physical and mental disorders. In other words, our results highlighted that the age cannot be considered a protective factor, with young women showed the same pattern as girls. This finding is relevant, suggesting that Italian female (in youth and/or emerging adulthood) adhere to thinness model of physical appearance at the expense of their own health (actual and future). Moreover, our findings is inline with Gribe et al. [1] suggestion ‘…in many ways, body dissatisfaction has emerged as core aspect of women’s physical and mental health….’ (p. 460).

In sum, this study clearly demonstrates that thinness model and adherence to it are strong factors influencing the perceived wellness of Italian girls and women, but likely cannot be considered a protective factor: thinness seemed to increase the vulnerability of youth for the development of disadaptive no toward to the healthy body management becoming a strong risk factor for future adulthood.

Our study has identified a severe phenomenon widespread among the Italians female population, highlighting the strong impact of sociocultural appearance on youth’s emotions but not their wellness attitudes. In fact, underweight girls tended to be more satisfied with their bodies but did not practice sports. Conversely, the overweight and obese girls exhibited stronger attitudes toward managing their own bodies and shapes to reinforce their own wellness. Although the normal distribution of girls into the BMI categories within the sample is representative and it is a positive data, normal BMI girls do not appear to engage in much healthy behaviour and their physical wellbeing appears to be mostly driven by the desire for social acceptance. This finding is contrary to that of Bearman et al. [19], who analysed the increasing body dissatisfaction among girls during growth and found that it was not strictly related to ideal body internalization, but rather to actual physical changes that deviated substantially from the social ideal.

The strengths of this study are the sample size and the homogeneous geographical area distribution of the sample, so it can be considered representative of the Italian female population in youth and emerging adulthood. The data draw a strong linkage between social appearance and body satisfaction, suggesting that dysfunctional body satisfaction are a strong risk factor for the healthy growth of the future adult generation.

The limitations of this study are related to the use of self-reports for psychological evaluation. Using a standardized psychological battery might provide a more objective in-depth assessment of personality and related dysfunctional affective and behavioural patterns.

## Conclusion

In conclusion, our results suggest that Girls and Young Women wish to be thinner, which leads them to neglect healthy behaviours. They prioritize social acceptance rather their own wellness and lifestyle quality. The underweight BMI class can be a considered a model to which girls adheres because it fits with the socially desired appearance. These findings can inform prevention and intervention efforts toward girl target and their health in order to reinforce their body satisfaction and behaviour by good practice, to ensure positive development and favour the awareness of health as own priority as well as own responsibility for future wellness: the self efficient body-management focused on own actual health and more in the expectation of future living should be boosted by the concept of ‘health as own responsibility’.

## Abbreviation

BMI: Body mass index
